# Internet- versus Telephone-based Local Outbreak Investigations

**DOI:** 10.3201/eid1406.071513

**Published:** 2008-06

**Authors:** Tista S. Ghosh, Jennifer L. Patnaik, Nisha B. Alden, Richard L. Vogt

**Affiliations:** * Tri-County Health Department, Greenwood Village, Colorado, USA; 1 Current affiliation: University of Colorado Health Sciences Center, Denver, Colorado, USA.; 2 Current affiliation: Boulder County Public Health, Boulder, Colorado, USA.

**Keywords:** Internet, disease outbreaks, local government, dispatch

## Abstract

We compared 5 locally conducted, Internet-based outbreak investigations with 5 telephone-based investigations. Internet-based surveys required less completion time, and response rates were similar for both investigation methods. Participant satisfaction with Internet-based surveys was high.

Although the Internet has been increasingly used in epidemiologic research, its use for investigation of infectious disease outbreaks has been less frequently described. Most reports of Internet-based outbreak investigations have described large, single outbreaks conducted by national or state public health agencies. Examples of reported Internet-based outbreak investigations include a communitywide norovirus outbreak in Finland, a communitywide *Cryptosporidium* outbreak in Kansas, a multistate *Salmonella* outbreak, and a conjunctivitis outbreak at a university (*1*–*4*). These reports noted several advantages of Internet use, including reductions in resource use, workload, and time required for survey completion and data entry (*1*–*4*). However, these advantages are not generally quantified in outbreak reports. Moreover, Internet-based outbreak investigations are seldom reported from the local health department level, where resources are often constrained compared with those of state and national agencies. We offer an analysis of several small Internet-based outbreak investigations conducted at the local level. We describe response rates to Internet-based surveys with and without telephone follow-up, the time needed to complete Internet-based outbreak surveys in comparison with traditional telephone surveys, participant satisfaction with Internet-based surveys, and differences in Internet-based outbreak investigations based on the respondents’ setting: professional versus household.

## The Study

From April through September of 2006, the Tri-County Health Department (TCHD), a local health department in metropolitan Denver, Colorado, used Internet-based surveys to investigate 5 outbreaks. Three outbreaks involved respondents in professional settings: a norovirus outbreak at a teacher appreciation luncheon, a norovirus outbreak at a catered professional meeting, and a norovirus outbreak at an office staff luncheon. The other 2 outbreaks involved respondents in household settings: a norovirus outbreak at a Father’s Day barbecue and a *Cryptosporidium* outbreak at a birthday pool party.

For all 5 outbreaks, a cohort study was conducted to ascertain illness and exposure information. Internet access among cohort members was assessed before Internet-based questionnaires were used. For 2 of the professional-setting outbreaks, all cohort members were sent by email a link to an Internet-based survey with directions on how to complete it and were asked to complete it by a certain deadline. In the third professional-setting outbreak, the office staff luncheon, the office requested that a link be sent to 1 office computer. Employees were individually given private access to that computer. In the 2 household-setting outbreaks, an email with a link to the Internet-based survey was sent to 1 household member, and household members took turns completing the survey. Cohort members of each outbreak were given the email address and telephone number of a TCHD contact, who was available to clarify survey-related questions. For all 5 outbreaks, an email reminder was sent out 1 day before the deadline. After the deadline had passed, follow-up telephone calls were made to nonresponders to improve response rates. Respondents were asked about their satisfaction with the Internet-based survey, as well as ease of use. Responses were entered directly into the Internet by the survey respondent, then downloaded by TCHD staff into Excel (Microsoft, Redmond, WA, USA) and analyzed with SAS (SAS Institute, Inc., Cary, NC, USA), thereby eliminating the need for data entry.

Response rates were then calculated, and the time required to complete each survey was tracked electronically. Response rates and survey completion time from the Internet-based investigations were then compared with those of 5 outbreaks in 2006 that were investigated by using traditional telephone surveys. All outbreaks in which TCHD conducted an epidemiologic investigation with a standardized questionnaire, from January through September 2006, were included in our study as either an Internet-based or telephone-based investigation. The questionnaires assessed illness-related symptoms, onset, and duration, as well as potential exposures. The number of questions ranged from 53 to 85 (median 67), depending on the number of possible exposures. The telephone-based investigations were similar to the Internet-based investigations in median sample size (Tables 1, 2), survey creation time, length, and content. Of TCHD employees who conducted all telephone interviews, 15 were surveyed to assess the time needed to complete the interviews. Statistical differences between the 2 investigation methods, for both median response rates and median survey completion time, were analyzed by using Wilcoxon rank-sum testing.

**Table 1:** Response rates for telephone-based outbreak investigations,  Denver, Colorado, 2006

Outbreak setting	N	Response rate, %
Elementary school	61	85
Italian restaurant	55	80
Childcare center	27	78
Japanese restaurant	14	86
Mother’s Day brunch	7	100

**Table 2:** Response rates for Internet-based outbreak investigations, by setting, Denver, Colorado, 2006

Outbreak setting	N	Response rate, %	
		Without follow-up telephone call	After follow-up telephone call
Professional			
Teacher appreciation luncheon	88	65	74
Office staff luncheon	20	60	95
Catered professional meeting	43	79	95
Household			
Father’s Day barbeque	15	100	100
Child birthday pool party	21	100	100

In this study, response rates to the Internet-based surveys were 60%–100%, before any telephone follow-up (Table 2). Response rates to the Internet-based surveys were lower in the professional-setting outbreaks but improved after follow-up telephone calls to nonresponders. In contrast, response rates in both household-setting outbreaks were 100% with the Internet survey alone; no follow-up telephone calls were necessary. No differences in completeness were found between those who took the survey online and those who completed the survey by telephone during a reminder follow-up call. In all 5 Internet-based investigations, most cohort members preferred the Internet-based survey to telephone or mailed surveys (online Appendix Table, available from www.cdc.gov/EID/content/14/6/975-appT.htm). Satisfaction with the Internet-based survey was lowest for those involved in the office staff luncheon outbreak (58%), for which employees took turns completing surveys on 1 computer. However, across outbreaks, most respondents (90%) found the Internet-based survey “very easy to use” (online Appendix Table, available from www.cdc.gov/EID/content/14/6/975-appT.htm).

Comparing the Internet-based investigations with the telephone-only investigations identified several differences. Telephone-based surveys took significantly more time to complete than Internet-based surveys (median of 30 min vs. 5 min, p<0.01). Median response rates to Internet-based surveys alone versus rates for telephone-based surveys alone were not significantly different (79% vs. 85%, p = 0.69). Median response rates improved when Internet-based surveys were combined with telephone follow-up; however, this improvement was not significantly different from telephone-only response rates (95% vs. 85%, p = 0.28).

## Conclusions

Local health departments often deal with constrained resources when investigating outbreaks. In this article, we have shown the utility of conducting Internet-based investigations at the local level. Telephone interviews are often the most time-consuming aspect of an outbreak investigation. In this study, a median time of 30 min was needed for health department employees to complete a telephone interview, whereas the Internet-based survey took a median of 5 min of the respondent’s time to complete and no health department employee time. Furthermore, fewer telephone calls were needed for the Internet-based investigations because follow-up telephone calls were made only to nonresponders, a small subset of cohort members. This was particularly true in the household-setting outbreaks, in which the response rate to the Internet-based survey was 100%, and no telephone calls were necessary. Moreover, because responses to the Internet-based survey were entered directly by the respondent, health department staff time for data entry was further reduced. Thus, Internet-based investigations can greatly reduce workload for local health departments. More studies are needed to further quantify the resource savings in Internet-based investigations and to analyze the effects of this data collection method on data quality.

We found that Internet-based surveys were associated with high levels of respondent satisfaction. Most respondents found the Internet-based survey preferable and easy to use. In professional-setting outbreaks, satisfaction may be higher when all participants have access to their own computers, rather than taking turns on a single computer. This finding contrasts with that of the household-setting outbreaks, in which, despite the sharing of a computer by household members, response rates and satisfaction rates were high. Thus, when Internet access is available, Internet-based surveys may be well received by respondents, particularly in household settings.

One limitation of this study is the small number of outbreaks analyzed and the small sample sizes. Another major limitation to any Internet-based outbreak investigation is participant access to the Internet. Internet-based investigations can only be used when Internet access is widespread among the group being surveyed. Factors such as age, race, and income level may affect the familiarity with and availability of the Internet, as may geographic and language-related issues (*5*).

Public health agencies should assess these factors before initiating an Internet-based outbreak investigation. When access issues are not present, the Internet can be a useful and economical tool for investigating outbreaks.
